# A Critical Examination of Subgroup Analyses: The National Acute Spinal Cord Injury Studies and Beyond

**DOI:** 10.3389/fneur.2018.00011

**Published:** 2018-02-01

**Authors:** Shannon Hextrum, Stephanie Bennett

**Affiliations:** ^1^Department of Neurology, Loyola University Medical Center, Maywood, CA, United States; ^2^Department of Pharmacy, Loyola University Medical Center, Maywood, CA, United States

**Keywords:** spinal cord injuries, subgroup analysis, statistics as topic, evidence-based medicine, methylprednisolone

## Abstract

The use of high-dose methylprednisolone for acute spinal cord injury continues to be a topic of debate. This controversy largely stems from fundamental issues in statistical interpretation of trial data, most notably subgroup analyses. The purpose of this review is to discuss important examples of improper subgroup analysis and encourage better practices in future research.

## Introduction

The National Acute Spinal Cord Injury Studies (NASCIS) of the 1980s and 1990s promoted the use of high-dose methylprednisolone in acute spinal cord injury but were widely criticized in subsequent years on aspects of methodology and statistical analysis (1–4). The methylprednisolone controversy sparked by NASCIS was recently addressed in a comprehensive review of acute spinal cord compression, thus highlighting the continued relevance of this issue (5). Improper and misleading subgroup analyses are fundamental errors in both NASCIS II and III. Unfortunately, issues in subgroup analysis are alarmingly common in the medical literature, likely contributing to the prolonged confusion and debate surrounding the NASCIS trials (4). It is worth revisiting the proper and improper use of subgroup analysis in order to provide tools for the neurologist to more clearly interpret NASCIS and critically assess future trials.

## Precedents in Subgroup Analysis

The appropriate use of clinical trial data in treating the individual patient has been an ongoing topic of concern in the medical community. Subgroup analysis has emerged as a potential solution, but must be approached with caution. Examples abound regarding the misuse and misapplication of subgroup analysis in clinical medicine (6). A 1978 study published in the *New England Journal of Medicine* used subgroup analysis to suggest that women with recent transient ischemic attacks (TIA) would not benefit from aspirin for stroke prevention (7). Based on this result, the FDA approved aspirin for stroke prevention after TIA in men only, until a revision in 1998 included women. Only 179 women were studied in this trial of 585 patients, demonstrating the study’s limited power for generalizing the subgroup results. However, this did not stop a widespread conclusion that women should not be given aspirin after TIA (8).

In 1988, the authors of the Second International Study of Infarct Survival (ISIS-2) made a point of examining the unreliability of subgroup analysis. Their primary study outcome supported a mortality benefit with both aspirin and streptokinase in acute myocardial infarction. The authors then subdivided the data by astrological sign, demonstrating that patients born as Gemini or Libra showed increased (though not statistically significant) mortality with aspirin (9). One would hope that this classic trial would encourage an immediate trend toward thoughtful subgroup analysis; however, the series of landmark NASCIS trials are evidence to the contrary.

## The NASCIS Trials

In 1984, Bracken et al. published NASCIS I, a study of 330 patients randomized to receive two different doses of methylprednisolone sodium succinate (MPSS) within 48 h of acute spinal cord injury (1). With results demonstrating no significant difference in motor recovery between groups, Bracken et al. concluded that the dosing of MPSS was not sufficient. As such, NACIS II, published in 1990, involved a MPSS treatment arm with a much higher, weight-based dose of MPSS compared to naloxone treatment and placebo (a placebo arm was conspicuously absent in NASCIS I). Similar to NASCIS I, the primary endpoint in NASCIS II was negative; however, the authors emphasized the positive results of a single, statistically significant subgroup of patients receiving MPSS within 8 h of injury (2). There are multiple issues with this subgroup analysis. First, the study was designed for patients to receive treatment within 12 h of injury, with no indications of other predetermined time intervals. As a *post hoc* analysis, the 8-h subgroup results should be a metric used for further study and should not have the same weight as the primary outcome. Second, the issue of data mining comes into play when considering the seemingly arbitrary cutoff of 8 h; one must assume that other timeframes were tested for statistical significance, and those intervals that were insignificant were never reported. For instance, there was likely a test of 0–3, 3–6, 6–9, etc., for all permutations within 12 h (4).

Transparency about the number of subgroups tested is one way in which NASCIS II may have been improved, yet, further steps must be taken to remedy the issue of multiple subgroups. This is the problem of multiplicity, meaning there is an increased probability of false positives when increasing the absolute number of subgroups analyzed (10). In fact, when 10 subgroups are analyzed, the probability of finding a statistically significant result due to chance alone is as high as 40%. One solution that has been suggested is to divide the alpha by the total number of subgroups; an alpha of 0.05 would change to 0.005 if 10 subgroups were analyzed (11). As previously discussed, NASCIS II never reported the total number of subgroups, so it is impossible to know the appropriate modified alpha for testing statistical significance.

The third and final NASCIS trial published in 1998 demonstrated similar issues in subgroup analysis. MPSS was administered for 24 or 48 h and compared to tirilazad mesylate, a lipid peroxidation inhibitor. Patients who received treatment within 8 h of injury, however, had results that were analyzed by various *post hoc*, time-to-treatment subgroups that were not delineated in the methods. Using these subgroup results, authors concluded the following: if bolus MPSS was given ≤3 h from injury, then dosing should continue for 24 h, and if given within 3–8 h of injury, MPSS should continue for 48 h (3). In response, spinal cord injury guidelines were adapted to recognize these timeframes (12).

## After NASCIS

The waning popularity of MPSS for acute spinal cord injury was not a result of another randomized controlled trial but was largely due to thoughtful reviews and critiques of the NASCIS trials. Unfortunately, as the era of evidence-based medicine has grown, issues of subgroup analysis are often overlooked. A 2007 report in the *New England Journal of Medicine* (NEJM) assessed the integrity of subgroup analysis in its journal during 1 year (July 1, 2005 to June 30, 2006). Out of the 97 trials published in NEJM, 59 (61%) reported subgroup analyses. Of those 59 studies, a total of 40 (68%) did not specify whether or not the subgroups were predetermined or *post hoc*. Nine trials (15%) did not specify the total number of subgroups examined, lending to the multiplicity issue discussed above (10). What’s perhaps more troubling was a 2011 study in the *British Medical Journal*, which examined subgroup analyses and sources of trial funding. In regards to trials without significant primary outcomes, the industry-funded trials more often reported subgroup analyses than those studies not funded by industry (13).

A more recent example of improper subgroup analysis is apparent in a 2008 follow-up analysis of the landmark ISTAT trial (International Subarachnoid Aneurysm Trial) (14). The ISTAT trial of 2002 reported morbidity and mortality benefit at 1 year for endovascular coiling over neurosurgical clipping of ruptured aneurysms (15). In contrast, the 2008 follow-up analysis concluded in favor of neurosurgical clipping for elderly patients with ruptured middle cerebral artery aneurysms. The ISTAT trial included 2,143 patients, and a pre-specified subgroup analysis based on age (i.e., <40, 40–49, 50–59, 60–69, ≥70 years) showed no trend in treatment effect by age. The 2008 report analyzed a *post hoc* subgroup of 278 patients ≥65 years, suggesting benefit to neurosurgical clipping of middle cerebral artery aneurysms (14, 15). Once again, a study with a small subgroup claiming results in the opposite direction of the primary outcome must be considered suspect. *Post hoc* subgroup analyses should be hypothesis generating, rather than mistaken for primary study results.

## Discussion

With the sheer volume of studies available in this era of evidence-based medicine, it is increasingly difficult to stay abreast of the literature, let alone take a discriminating view of the statistical issues within it. The fundamental subgroup problems in the NASCIS trials are easy to overlook on a preliminary read. The simplest solution to avoid errors in subgroup interpretation is for authors to draw attention to their own use of subgroups, highlighting possible pitfalls. A clear, albeit extreme, example was seen in the ISIS-2 trial subgroup analysis based on astrologic signs (9). Nevertheless, a simple qualifying statement about subgroup effects is sufficient to remind the reader about the likelihood of false positives (i.e., the issue of multiplicity). It is also critical that studies are forthcoming with the number of subgroups tested and whether or not those subgroups were predetermined.

In summary, the reliability of subgroup analysis depends on whether the groups are predetermined, powered correctly, and corrected for issues such as multiplicity. The good clinician should approach every subgroup analysis with the attitude that not all statistically significant results are statistically sound.

## Author Contributions

SH conceived the idea. SH and SB drafted the manuscript.

## Conflict of Interest Statement

The authors declare that the research was conducted in the absence of any commercial or financial relationships that could be construed as a potential conflict of interest.
